# Role of the Wnt/*β*-Catenin Pathway in Renal Osteodystrophy

**DOI:** 10.1155/2018/5893514

**Published:** 2018-04-02

**Authors:** Sarah-Kim Bisson, Roth-Visal Ung, Fabrice Mac-Way

**Affiliations:** Faculty and Department of Medicine, CHU de Québec Research Center, L'Hôtel-Dieu de Québec Hospital, Endocrinology and Nephrology Axis, Université Laval, Quebec, Canada

## Abstract

Vascular calcification and bone fragility are common and interrelated health problems that affect chronic kidney disease (CKD) patients. Bone fragility, which leads to higher risk of fracture and mortality, arises from the abnormal bone remodeling and mineralization that are seen in chronic kidney disease. Recently, sclerostin and Dickkopf-related protein 1 were suggested to play a significant role in CKD-related bone disease as they are known inhibitors of the Wnt pathway, thus preventing bone formation. This review focuses on new knowledge about the Wnt pathway in bone, how its function is affected by chronic kidney disease and how this affects bone structure. Expression of components and inhibitors of the Wnt pathway has been shown to be affected by the loss of kidney function, and a better understanding of the bone effects of Wnt pathway inhibitors could allow the development of new therapies to prevent bone fragility in this population.

## 1. Introduction

Chronic kidney disease-mineral and bone disorder (CKD-MBD) is a major health issue as it is associated with increased bone fracture risk and development of vascular calcification [1]. In recent years, studies have suggested that the Wnt/*β*-catenin pathway plays a potential and promising role in CKD-MBD, as the activation of this pathway increases bone formation and Wnt inhibitor levels were shown to be increased in CKD. Studies have then focused on understanding how Wnt inhibitors such as sclerostin and Dickkopf-1 (Dkk1) were involved in the development of bone turnover anomalies and vascular calcification in CKD [2–4]. As of now, the reason why Wnt inhibitor levels are increased in CKD, as well as their exact role in inducing or preventing anomalies of bone turnover, still remains poorly understood. A better comprehension of the Wnt/*β*-catenin pathway will provide new perspectives for understanding the pathophysiology of CKD-MBD and may pave the way for the development of new targeted treatments.

The aim of this review is to discuss the current knowledge on (1) Wnt/*β*-catenin pathway function and regulation in bone metabolism, (2) the implication of Wnt inhibitors in the development of CKD-related bone anomalies, and (3) the potential benefits of inhibiting Wnt inhibitors to improve bone disease in CKD. We decided to focus our review on bone disease as other excellent review papers already exist on the role of the Wnt/*β*-catenin pathway in vascular calcification.

## 2. Mineral Abnormalities in CKD

**Figure 1:**
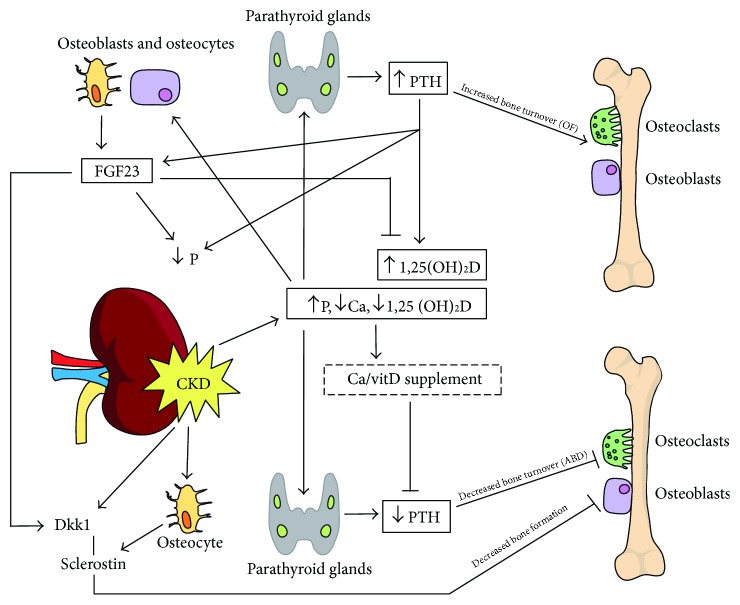
Mineral and bone disorders in chronic kidney disease (CKD). Elevated phosphorus (P), low calcium (Ca), and 1,25 (OH)_2_D (vitD) stimulate parathyroid hormone (PTH) production by the parathyroid glands, which leads to increased bone turnover and P excretion by the kidneys. High PTH and P also seem to either directly or indirectly stimulate fibroblast growth factor 23 (FGF23) production by osteoblasts and osteocytes, though this seems at least partly mediated by an upregulation of vitD by high PTH, which in turn activates FGF23 production. While FGF23 stimulates P excretion, it further suppresses vitD production. Suppression of PTH levels may arise from Ca and vitD supplements or treatment with calcimimetics in CKD. Suppressed PTH levels will lead to decreased bone turnover.

Bone turnover is the result of a tight coordination between bone formation by osteoblasts (OB) and bone resorption by osteoclasts (OC). Osteoclast activity depends on the balance between receptor activator of nuclear factor kappa-B ligand (RANKL) produced by the osteoblasts, which increases OC proliferation and differentiation, and osteoprotegerin (OPG), which binds to RANKL to inhibit osteoclastic activation [5]. The osteocytes, which are the ultimate differentiation stage of osteoblast-lineage cells and the main cellular components of bone, also act as regulators of bone turnover. Indeed, osteocytes allow the bone to adapt to new conditions by variably expressing OPG, RANKL [6], and other proteins that act on OB and OC. In CKD, dysregulation of parathyroid hormone (PTH), which modulates OPG and RANKL, has considerable repercussions on bone turnover (Figure 1). Hypocalcemia develops as a result of decreased renal production of 1,25 (OH)_2_D, which is necessary for the intestinal absorption of calcium. Concurrently, hyperphosphatemia occurs due to the inability of the failing kidneys to excrete excess phosphorus. Hypocalcemia, hyperphosphatemia, and decreased 1,25 (OH)_2_D all contribute to the development of secondary hyperparathyroidism, which is meant to normalize levels of serum calcium, phosphate, and 1,25 (OH)_2_D through its effects on the bone and kidney [7]. If untreated, secondary hyperparathyroidism leads to an increased bone turnover in favor of bone resorption and bone loss [8]. At a very early stage of CKD (glomerular filtration rate < 60–70 ml/mn/1.73 m^2^), there is elevation of fibroblast growth factor 23 (FGF23), a phosphaturic hormone secreted by the osteocytes that also inhibits 1,25 (OH)_2_D production [9]. The precise causes of its elevation remain unclear, but it seems to be mainly related to 1,25 (OH)_2_D, hyperphosphatemia, calcium, PTH, and metabolic acidosis [10]. Recent studies also suggest that iron deficiency is associated with high FGF23 levels in a rat model of CKD [11].

### 2.1. Types of Bone Diseases in CKD

Renal osteodystrophy is defined by the abnormalities of bone turnover, mineralization, and microarchitecture that affect CKD patients. Classically, four types of bone diseases specific to CKD are defined:

1. *Hyperparathyroidism bone disease* is the classical high bone turnover disease that is mainly due to untreated or undertreated secondary hyperparathyroidism. Bone anomalies are characterized by thinning of the cortical bone and accumulation of abnormal trabecular bone [12].
2. *Adynamic bone disease (ABD)*, characterized by low or absent bone formation and resorption, is a common finding in early CKD and especially in predialysis CKD patients with diabetes [13, 14]. The aetiology of ABD is currently unknown but has been associated with oversuppression of PTH levels and development of PTH resistance [14]. Studies have suggested that patients with low PTH levels are more susceptible to fractures [15, 16].
3. *Osteomalacia (OM)*, defined by slower bone turnover and accumulation of nonmineralized bone matrix, leads to reduced bone strength. In CKD, OM is often secondary to vitamin D deficiency, uncontrolled metabolic acidosis, or hypocalcemia, but the exact aetiology is often unknown [17].
4. *Mixed renal osteodystrophy* is characterized by a combination of high bone turnover disease and mineralization defects [14].

## 3. Overview of Wnt/*β*-Catenin Pathway

The Wnt/*β*-catenin pathway has been recognized as a major regulator of bone formation. Its activation stabilizes *β*-catenin, a transcription factor that stimulates the production of osteoblastic transcription factors such as Runt-related transcription factor 2 (Runx2) and osterix. Wnt/*β*-catenin pathway activators such as the Wnt ligands thus raise the number of mineralizing osteoblasts and increase the rate of bone formation [18, 19]. The following section will describe the current knowledge on the role of Wnt/*β*-catenin pathway in the regulation of osteoblastogenesis.

### 3.1. Osteoblasts, Wnt/*β*-Catenin Pathway, and Regulation of the Bone Turnover

**Figure 2:**
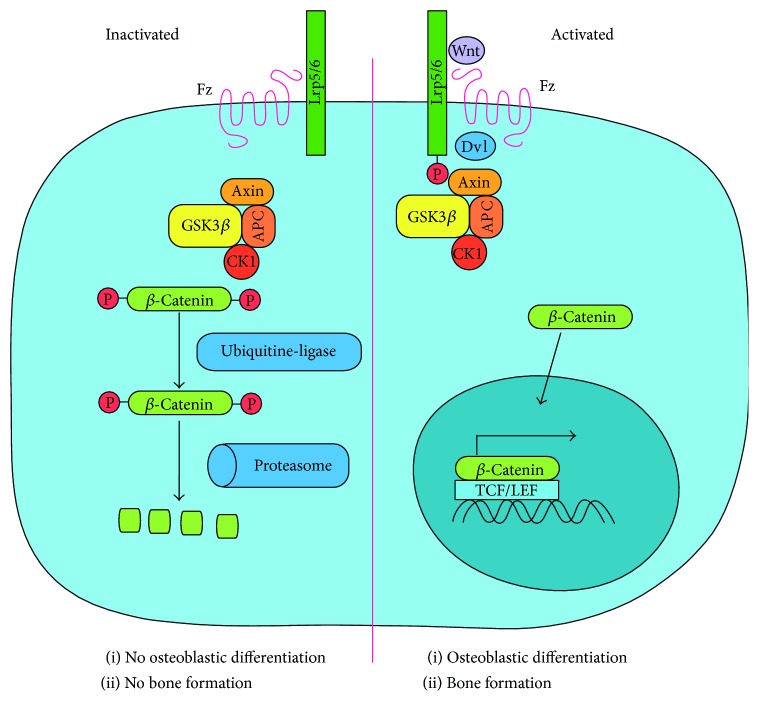
Wnt/*β*-catenin pathway and bone formation. On the left, no Wnt ligand is present and the phosphorylation complex, which consists of GSK3*β*, APC, CK1, and Axin, can freely phosphorylate *β*-catenin. This phosphorylation signals to an ubiquitin ligase that *β*-catenin has to be marked by degradation by the proteasome. On the right, a Wnt ligand binds to Frizzled (Fz) receptor and Lrp5/6 coreceptor. This causes Dvl to phosphorylate Lrp5/6, leading to the recruitment and subsequent inactivation of the phosphorylation complex. *β*-Catenin is free to translocate to the nucleus and act as a transcription factor for osteoblastic genes.

The first step in the activation of this pathway is the binding of a Wnt ligand from the extracellular environment to two transmembrane proteins: a Frizzled protein (Fz) acting as the receptor and a low-density lipoprotein receptor-related protein 5 or 6 (Lrp5/6) acting as a coreceptor (Figure 2). Wnt1, Wnt3a, and Wnt10b seem to be the main Wnt ligands that activate osteoblastic differentiation [19]. Upon binding of one of these ligands, Fz and Lrp5/6 are brought together [20]. This causes the recruitment of the protein Disheveled (Dvl), which phosphorylates Lrp5/6. The phosphorylation of the coreceptor leads to the recruitment of Axin, an essential component of the *β*-catenin phosphorylation complex [21-23]. This complex is necessary for the degradation of *β*-catenin and is composed of three other proteins: glycogen synthase kinase 3 beta (GSK3B), adenomatous polyposis coli (APC), and casein kinase I (CKI). Following the recruitment of Axin to Lrp5/6, the *β*-catenin phosphorylation complex binds to Dvl and is destabilized. Dvl blocks the phosphorylation of Axin by GSK3*β*, which is necessary for phosphorylation of *β*-catenin by the complex [23]. The unphosphorylated *β*-catenin is then free to translocate to the nucleus, interact with a T cell factor/lymphoid enhancer factor (Tcf/Lef) element on the DNA, and initiate the transcription of genes involved in osteoblast differentiation. As demonstrated with the use of a GSK3*β* inhibitor in mice, which inactivates the *β*-catenin phosphorylation complex, activation of the Wnt/*β*-catenin pathway results in increased bone mass [24]. Apart from genes involved in osteoblastic differentiation, activation of the Wnt/*β*-catenin pathway results in upregulation of other genes. Among them is Axin2, a protein that can play a similar role as Axin in the phosphorylation complex. Axin2 is however less susceptible to Dvl regulation than the constitutively expressed Axin, which means that Axin2-containing complexes tend to be less efficient at activating *β*-catenin signaling despite Wnt activation [25]. This suggests that Axin2 could be part of a negative feedback mechanism that makes cells less sensitive to Wnt ligands without altogether turning off the Wnt/*β*-catenin pathway. In the absence of Wnt ligands or in the presence of Wnt inhibitors (see related section below), there is a reduced activation of the Wnt/*β*-catenin pathway. Since the phosphorylation complex is not recruited to Lrp5/6 and Dvl, it will phosphorylate *β*-catenin in multiple locations through the action of GSK3B and CK1 (Figure 2). These phosphate groups then allow an E3 ubiquitin ligase to mark the *β*-catenin molecule for degradation by the proteasome [26]. The ensuing reduction in *β*-catenin levels translates to decreased transcription of osteoblastic differentiation factors, thus reducing bone formation. In addition to its effect on bone formation, studies have shown that activation of the Wnt/*β*-catenin pathway in osteoblasts was also associated with decreased levels of RANKL and increased levels of OPG, which lead to suppressed bone resorption in mice and lower production of resorption markers in cultured osteoblasts [27, 28]. Conversely, osteoblast-specific deletion of *β*-catenin was shown to decrease the levels of OPG [29] and to increase RANKL levels, which result in increased bone resorption and deficient mineralization [28]. Production of Wnt activators and inhibitors is therefore a way of balancing bone formation and resorption.

### 3.2. Osteocytes, Wnt/*β*-Catenin Pathway, and Regulation of the Bone Turnover

Osteocytes, which originate from terminally differentiated osteoblasts entrapped in the bone matrix they produced, are the main source of Wnt inhibitors sclerostin and Dkk1 in the bone [30, 31]. They have the capacity to communicate with osteoblasts, osteoclasts, and other osteocytes through cytoplasmic prolongations. At the end of these dendrites are gap junctions that allow the osteocytes to obtain nutrients and transmit molecular signals to neighboring cells [32]. Recently, it has been recognized that osteocytes play a major role in the bone mechanotransduction. Indeed, osteocytes form a complex network that senses changes in bone loading and produces proteins that affect osteoclastic and osteoblastic activity. Mechanical unloading of the bone stimulates the production of Wnt inhibitors such as sclerostin by osteocytes and RANKL by osteoblasts in the “unloaded” region, resulting in decreased activation of the Wnt/*β*-catenin pathway in neighboring cells and increased bone resorption [6, 33]. In contrast, during mechanical loading, the osteocyte network produces less sclerostin, resulting in activation of Wnt/*β*-catenin signaling in preosteoblasts to increase bone formation [33, 34]. Therefore, the osteocyte network does not only regulate its own production of sclerostin in response to loading but also osteoblastic production of RANKL, which regulates osteoclastic activity. Moreover, activation of the Wnt/*β*-catenin pathway in osteocytes themselves also results in modification of the RANKL/OPG ratio. Indeed, previous studies have reported that mice lacking *β*-catenin in osteocytes had more osteoclasts and lower bone mass due to decreased osteocytic RANKL/OPG ratio [35, 36], which is consistent with what was observed in osteoblasts. Conversely, Li et al. found that a constitutive activation of osteocytic *β*-catenin instead led to a decreased RANKL/OPG ratio [36]. Another study reported different results as overexpression of *β*-catenin in osteocytes stimulated bone resorption by raising the RANKL/OPG ratio [27]. However, this last observation seems to be true only when sclerostin is also elevated in the osteocytes.

### 3.3. PTH Affects the RANKL/OPG Ratio through Wnt/*β*-Catenin Pathway

**Figure 3:**
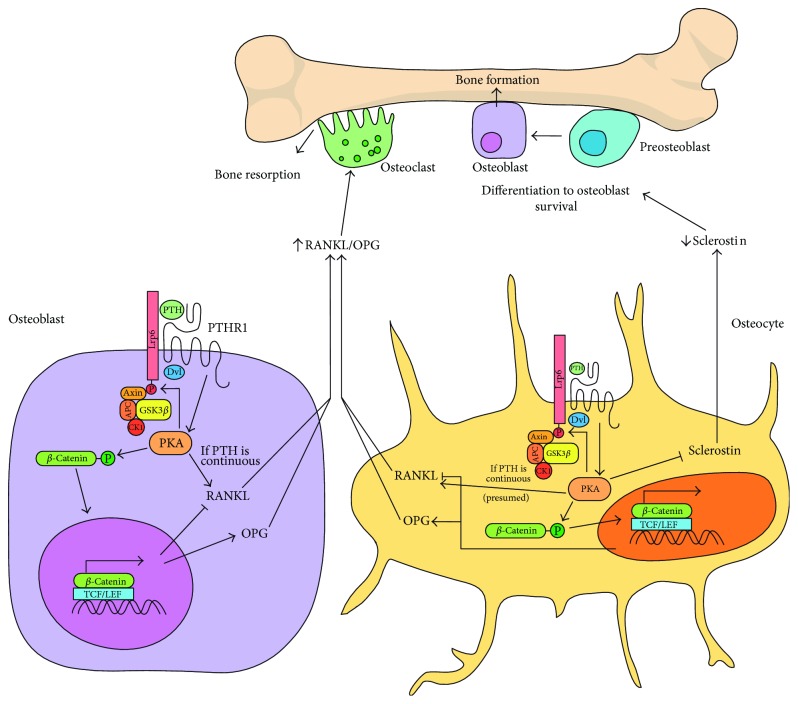
Proposed mechanisms by which PTH activates Wnt/*β*-catenin pathway in osteoblasts and osteocytes to modulate bone turnover. In the osteoblast (left), PTH binding to Lrp6 and PTH1R activates PKA, which activates the Wnt/*β*-catenin pathway by phosphorylating Lrp6. The levels of *β*-catenin rise, which leads to modulation of RANKL and OPG expression resulting in a decreased RANKL/OPG ratio. PKA itself can also directly stabilize *β*-catenin through phosphorylation and also directly stimulates RANKL production. The increased production of RANKL when PTH is administered continuously might be mediated by this action of PKA. A similar mechanism occurs in the osteocyte (right), presumably also through Lrp6 signaling. While it is unconfirmed whether PKA can also directly stimulate RANKL transcription in osteocytes, continuous PTH in osteocytes activates RANKL production while only activating the Wnt/*β*-catenin instead increases OPG production. A notable difference between how both cell types respond to PTH is that PKA activation inhibits sclerostin production in osteocytes. The decrease in sclerostin could partly explain the resulting increase in bone formation when PTH is given intermittently. Moreover, expression of sclerostin in osteocytes seems to further upregulate RANKL in those same cells.

Continuous secretion of PTH is associated with increased bone turnover in favor of bone resorption through upregulation of RANKL and downregulation of OPG [37, 38]. However, when given intermittently to patients for a short period of time, PTH increases bone turnover in favor of bone formation and is currently used as a treatment for osteoporosis [38, 39]. The mechanisms by which PTH regulates RANKL and OPG production in the bone are not completely understood, but the Wnt/*β*-catenin pathway is likely to be involved. PTH can bind to its receptor PTH1R and the Wnt coreceptor Lrp6 on the surface of osteoblasts, which results in *β*-catenin stabilization despite no Wnt ligand being involved (Figure 3). This is explained by the fact that PTH binding activates protein kinase A (PKA), which directly activates osteoblastic RANKL transcription, stabilizes *β*-catenin, and also phosphorylates Lrp6 [40-42]. Following Lrp6 phosphorylation by PKA, Axin is recruited and *β*-catenin is further stabilized, as would be observed if the Wnt/*β*-catenin pathway was activated by a Wnt ligand [40]. Since a rise in *β*-catenin levels has been associated with decreased RANKL and increased OPG, this may partly counter the upregulating effect of PKA on RANKL [28, 42]. Osteoblastic Lrp6 is required for PKA activation and RANKL upregulation in mice in response to PTH, suggesting that its paralog Lrp5 cannot compensate for its absence [40, 42]. PTH also seems to regulate RANKL production in osteocytes. In a study by O'Brien et al., activation of PTH1R in the osteocytes of mice led to increased bone turnover and bone mass, the first being explained in part by an increase in RANKL expression and the latter by a suppressing effect of PTH on Wnt inhibitor sclerostin, which leads to increased Wnt/*β*-catenin activity in bone cells [43, 44]. It seems that Lrp5 is necessary for the action of sclerostin, since system-wide deletion in mice prevents the bone anabolic effect of PTH1R activation in osteocytes [43]. PKA could be responsible, at least in part, for the increased RANKL through direct upregulation in osteocytes, as was the case in osteoblasts, but the Wnt/*β*-catenin pathway could also contribute to the RANKL/OPG ratio. Direct upregulation of *β*-catenin in osteocytes has been reported to increase OPG in mice as observed in osteoblasts, so it is likely that the downregulation of osteoblastic RANKL by *β*-catenin could also be observed in osteocytes [29].

### 3.4. Lrp5/6 Mediates the Effects of PTH on Osteoblasts and Osteocytes

Lrp6 seems to be necessary for activation of PTH target genes in osteoblasts. Indeed, deletion of Lrp6 prevents both the increased bone formation and resorption that normally result from PTH1R activation in osteoblasts [42]. Lrp6 also seems more important for regulation of bone turnover than Lrp5, as its deletion appears more deleterious to trabecular bone structure and osteoblastic differentiation than that of Lrp5 [45]. This could be explained by the fact that Lrp6, unlike Lrp5, is phosphorylated by PKA following PTH binding, which is a necessary step for *β*-catenin stabilization in response to PTH [40]. However, Lrp5 still seems to be involved in the anabolic effects of PTH, as its global deletion prevents bone formation when PTH1R is activated in the osteocytes of mice without impacting RANKL production [43]. Since global deletion of Lrp5 does not prevent the anabolic effects of intermittent PTH injections [46, 47], it is possible that the only role of Lrp5 in the PTH anabolic response is to mediate the effects of sclerostin on osteoblasts.

## 4. Regulation of Bone Metabolism by Wnt/*β*-Catenin Pathway Inhibitors

**Figure 4:**
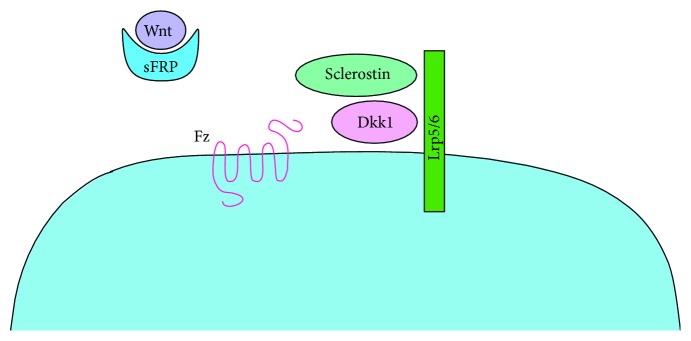
Mechanisms of action of common Wnt inhibitors. Sclerostin (light green) and Dkk1 (light pink) both prevent Wnt/*β*-catenin pathway activation by binding to Lrp5/6, which prevents its interaction with Frizzled (Fz). The primary mechanism of action of SFRPs (blue) is to bind to Wnt molecules thus preventing its interaction with its receptor and further activate the Wnt/*β*-catenin pathway.

Wnt inhibitors such as sclerostin, Dkk1, and secreted frizzled-related proteins (SFRPs) are produced by the osteocytes and prevent Wnt/*β*-catenin pathway activation by two main mechanisms: through competitive binding on its receptor or through their binding to the Wnt ligands (Figure 4) [30]. Wnt inhibitors are therefore highly involved in the coordination of bone formation and resorption. The following sections are meant to explain the current knowledge on the role of these inhibitors in the regulation of bone turnover.

### 4.1. Sclerostin and Regulation of Bone Turnover

Unlike the other Wnt inhibitors, sclerostin is often thought of as a specifically osteocytic protein [48]. By binding to Lrp5/6, sclerostin prevents the activation of the Wnt/*β*-catenin pathway in response to the binding of a Wnt ligand. In humans, mutations of the sclerostin gene, SOST, are known to have adverse effects on bone. These mutations, whether they are nonsense, missense, or other, all prevent the secretion of functional sclerostin and typically lead to thicker cortical bone [49]. In humans, the effects are particularly visible in the skull, where the excess bone can crush facial nerves and increase intracranial pressure [50, 51]. In mice, inactivation of SOST leads to increased bone mass through increased activity of the Wnt/*β*-catenin pathway [52] while activating mutations lead to bone loss [53]. The use of antibodies against sclerostin in animal studies supports these results, since this treatment has resulted in greater bone mass and faster fracture repair as compared to controls [54-56]. In addition to inhibiting osteoblastic differentiation through the Wnt/*β*-catenin pathway, sclerostin has been shown to negatively regulate the bone morphogenetic protein (BMP) pathway, which is also involved in bone formation [57]. Furthermore, high levels of sclerostin may potentially induce bone mineralization defects as it normally downregulates PHEX (phosphate-regulating neutral endopeptidase), a peptide responsible for maintaining low levels of bone mineralization inhibitor MEPE-ASARM (matrix extracellular phosphoglycoprotein with an acidic-serine and aspartate-rich motif) [58]. In addition to inhibition of bone formation, recent studies have shown that sclerostin might also be an activator of bone resorption by increasing RANKL/OPG ratio in osteocytes [27, 59]. Moreover, sclerostin has been shown to interact with more than a dozen other bone proteins in affinity capture studies, suggesting it could also modulate bone metabolism through other pathways than Wnt/*β*-catenin [60]. Binding interactions have been reported with the enzyme alkaline phosphatase, the Phex endopeptidase, and the Wnt antagonist SFRP4, but the biological meaning of most of these interactions remains undiscovered [60]. The demonstration that sclerostin has the ability to interact with a wide variety of proteins and that its expression range also includes the heart, kidneys, liver, and lungs reveals the complexity of its role, which is probably not only limited to bone regulation [61]. More recently, Lrp4, which resembles the Wnt coreceptors Lrp5/6 in structure, has been established as an important mediator of the effects of sclerostin. For more than ten years, it has been known that Lrp4 acted as a Wnt inhibitor because of the bone phenotype resulting from its deletion or mutation. These include syndactyly and cranial deformation in mice, cattle, and humans, which are reminiscent of diseases caused by a mutated SOST gene [62-64]. Moreover, cases of sclerosteosis in humans, which are often the result of mutations of the SOST gene, have been attributed to similar disruptions of the LRP4 gene [50]. While the mechanism by which Lrp4 causes Wnt inhibition has remained unclear for years, the implication of the receptor in the development of neuromuscular junctions has been much studied and abundantly described [65, 66]. When targeted by autoantibodies, Lrp4 cannot bind its ligand agrin, which results in faulty neuromuscular junction development that leads to a serious disease called myasthenia gravis [67]. It is only recently that a satisfying explanation has been brought up for the link between Lrp4 mutations and high bone mass. While Lrp4 cannot activate the Wnt pathway like Lrp5/6, it can nonetheless bind sclerostin and has thus been posited to act as an anchor for the protein in bone [68]. This would explain the fact that while Lrp4 deficiency leads to higher serum sclerostin levels, the bone production of sclerostin does not appear increased, Wnt is more activated in osteoblasts, and bone mass increases as a result [69-71]. Lrp4 deletion also leads to decreased RANKL and Dkk1 production, as seen when the Wnt/*β*-catenin pathway is active [70, 72]. This is further supported by the fact that deletion of the sclerostin-binding domain of Lrp4, or use of anti-Lrp4 antibodies, leads to increased bone formation [71, 73].

### 4.2. Dkk1 and Bone Formation

While Dkk1 is highly expressed in osteocytes similarly to sclerostin, it is also produced by osteoblasts and cells involved in embryogenesis [74]. It binds to Lrp5/6 to inhibit Wnt/*β*-catenin pathway signaling and thus bone formation. Overexpression of Dkk1 in mice has been associated with decreased bone mass and bone formation through inhibition of the Wnt/*β*-catenin pathway, similar to what is observed when sclerostin is overexpressed [75]. Moreover, constant Dkk1 activation seems to prevent PTH-induced elevation of bone turnover [76]. One allele deletion of the Dkk1 gene is sufficient to induce a marked increase in bone formation in rats [77], but the effects of complete deletion are more complex to study since such mutation prevents animals from reaching the end of their development. Nonetheless, inhibition of Dkk1 using monoclonal antibodies has resulted in increased bone formation, as seen with sclerostin inactivation [78]. Despite Dkk1 also having the capacity to bind Lrp4, it is currently unknown whether or not its levels are affected by Lrp4 deletion [68].

### 4.3. SFRPs and Bone Formation

SFRP-1, SFRP-2, and SFRP-4 are produced by a greater variety of cells, including osteoblasts, and seem to have a similar effect on bone as sclerostin and Dkk1 [79]. Their mechanisms of action are however slightly different, since SFRPs normally bind to the Wnt ligands in a decoy receptor fashion [79]. Consistent with what has been observed in cases of increased sclerostin and Dkk1 expression, high SFRP1 expression was shown to be correlated with preosteocytic apoptosis in vitro, whereas SFRP1 deletion in vivo increased osteoblastic survival and trabecular bone volume [80]. Interestingly, by directly binding RANKL, SFRP1 also inhibits osteoclastogenesis [81]. In addition to its inhibitory effect on the Wnt/*β*-catenin pathway, SFRP4 has previously been shown to increase urinary phosphate excretion when given to parathyroidectomized rats, but a more recent study in which SFRP4 was ablated in mice did not show an effect on phosphorus metabolism, suggesting that the phosphaturic effect might not be observable at normal PTH levels [82, 83].

## 5. Determinants of Wnt Inhibitor Levels

Apart from mechanical loading, there are many other factors that can stimulate the production of Wnt inhibitors from osteoblasts and osteocytes. The next section summarizes some of the important factors that have been shown to influence sclerostin, Dkk1, and SFRPs levels.

### 5.1. PTH Regulates Wnt Inhibitors

PTH is a well-known inhibitor of sclerostin expression in osteocytes [84]. This action probably mediates, at least partially, the anabolic effect of PTH on bone. The exact mechanism behind this inhibition is not well defined, but it might be similar to the upregulation of RANKL, which results from PKA activation. Indeed, it has been determined that sclerostin is a direct target gene of PKA [44]. PTH has also been reported to influence the production of Dkk1 through an unknown mechanism. When PTH was added to cultured preosteoblastic cells or injected to parathyroidectomized rats, Dkk1 levels were decreased [76, 85]. Conversely, it has been reported that postmenopausal women with primary hyperparathyroidism have higher serum Dkk1 levels than controls [86]. It is currently unknown whether PTH has a significant effect on the regulation of Dkk1 levels in normal physiology.

### 5.2. Age and Gender Affect Dkk1 and Sclerostin Levels

Notably, age and gender have been associated with differences in sclerostin and Dkk1 levels. Women tend to have lower serum sclerostin and higher Dkk1 levels than men, while both serum sclerostin and Dkk1 levels have been reported to increase with age [87, 88].

### 5.3. Vitamin D Regulates Sclerostin Levels

In healthy men, supplementation of vitamin D and calcium has been associated with higher sclerostin levels, and observations supporting direct stimulation of sclerostin production by 1,25 (OH)_2_D have been made in cultured primary osteocytes [89, 90]. In contrast, in the case of vitamin D-deficient women treated with vitamin D_3_, higher serum levels of 25 (OH) D have been associated with lower sclerostin levels, indicating that health status might affect the regulation process [91].

### 5.4. BMP Regulates Sclerostin and Dkk1

Activation of the BMP pathway through upregulation of BMP receptors in osteoblasts leads to higher levels of both sclerostin and Dkk1 [92]. Since sclerostin has been shown to act as a BMP signaling antagonist in osteocytes, this suggests that sclerostin is part of a negative feedback loop aimed at reducing its own production [57].

## 6. FGF23 Regulates Bone Metabolism

FGF23 normally binds to FGFR and its coreceptor *α*-klotho in order to induce changes in the kidney, the parathyroid gland, and the bone. The extracellular part of *α*-klotho can also be cleaved and enter the circulation, where it mediates FGF23 action on other organs [93]. More recently, FGF23 and its coreceptor *α*-klotho have been shown to directly stimulate Dkk1 and sFRP1 production in cultured osteoblastic rat cells, adding to the list of osteocytic proteins that influence bone turnover through Wnt/*β*-catenin pathway inhibition. Indeed, the binding of FGF23 to its receptor FGFR and the secreted form of its coreceptor klotho on osteoblasts activates mitogen-activated protein kinase (MAPK) pathway, which leads to increased Dkk1 and sFRP1 production. While increased Dkk1 levels were associated with a decrease in *β*-catenin levels, sFRP1 did not seem to have the same effect [2]. Through their action on Dkk1, FGF23 and *α*-klotho can therefore be considered as indirect Wnt inhibitors. Due to its important role in CKD (see section below), we will explain in the next section the effects of FGF23 on the kidney and the parathyroid gland.

### 6.1. Effects of FGF23 on the Kidney

FGF23 is widely recognized to increase phosphorus excretion by the kidney, decreasing the number of type IIa sodium-phosphate cotransporters in the proximal tubule [94]. FGF23 also inhibits the production of 1,25 (OH)_2_D by downregulating 1-*α*-hydroxylase, which hinders the intestinal absorption of calcium and phosphorus [95]. Mice with the absence of FGF23 develop vascular calcification secondary to elevated phosphorus and 1,25 (OH)_2_D levels [96].

### 6.2. PTH and FGF23/Klotho Axis

Another important target organ of FGF23 is the parathyroid gland, where it suppresses PTH production. This inhibition seems to involve its binding to FGFR and *α*-klotho, followed by the activation of the MAPK pathway, as demonstrated by loss of PTH inhibition when the pathway was blocked in rats [97]. FGF23 also stimulates the production of *α*-klotho in the parathyroid gland, which could be expected to reinforce the inhibition of PTH [97]. Interestingly, PTH also appears to have a regulatory action on FGF23, upregulating its bone production [98]. In this regard, osteocytes seem to play a major role since activation of PTH1R in these cells significantly raises osteocytic FGF23 expression in mice [99]. This stimulatory effect of PTH probably involves the Wnt/*β*-catenin pathway since concomitant upregulation of sclerostin prevents the rise of FGF23 levels and *β*-catenin upregulation alone increases FGF23 [99]. Moreover, PTH also upregulates renal production of *α*-klotho [100]. The effects of PTH on FGF23 in the bone and on *α*-klotho in the kidney are thus a part of a negative feedback loop.

### 6.3. Vitamin D and Phosphorus Regulate FGF23

While FGF23 has a potent inactivating effect on 1-*α*-hydroxylase, 1,25 (OH)_2_D also regulates FGF23 production in vivo and in vitro [101-103]. This action is independent of PTH, as it has been observed in parathyroidectomized rats [100, 103]. In fact, it is thought that part of the action of PTH on FGF23 is through 1,25 (OH)_2_D upregulation, and an in vitro study has even reported no effect of PTH alone on FGF23 levels in osteoblastic cells [100, 102]. Interestingly, phosphorus does not seem to have a direct influence on FGF23 levels despite the fact that the two often correlate [101, 102]. Instead, it has been suggested that PTH is required to mediate phosphorus action on FGF23, as supported by the fact that parathyroidectomized rats have low FGF23 levels despite high phosphorus levels [100]. The effect of phosphorus on FGF23 also appears to be tied to 1,25 (OH)_2_D as phosphorus can synergistically increase the effect of 1,25 (OH)_2_D on FGF23, but does not have any effect in the absence of a functional vitamin D receptor [101, 104].

### 6.4. Treatment to Reduce FGF23 Levels

FGF23 has been targeted directly with antibodies in CKD rats with hyperparathyroidism, but unfortunately, the results showed a worsening of hyperphosphatemia and vascular calcification [105]. The increased mortality rates that resulted from this study raised the concern that treatments aiming to reduce FGF23 could do more harm than good especially if phosphorus is not kept within normal values.

## 7. Role of Wnt Inhibitors in the Development of CKD-MBD

Recent studies have associated anomalies of the Wnt/*β*-catenin pathway with bone disorders and vascular calcification in CKD, thus potentially contributing to CKD-MBD. This section will explain how Wnt inhibitors may negatively affect bone metabolism in CKD.

### 7.1. CKD Alters the Production of Wnt Inhibitors

A number of studies have now reported an increase in sclerostin, Dkk1, SFRP1, and SFRP4 levels as CKD progresses [2, 106, 107]. Notably, sclerostin levels increase in CKD despite higher renal elimination and have been associated with adynamic bone disease [2, 3, 9, 107–109]. Until now, the reason why Wnt inhibitors are elevated in CKD is unclear while some specific factors related to CKD may influence their levels.

### 7.2. Serum Phosphorus Is Associated with Wnt Inhibitors Levels

High serum phosphorus levels are a hallmark of CKD and are potentially the first responsible for increased Wnt inhibitors [110]. Indeed, serum sclerostin and DKK1 are positively correlated with phosphorus levels in CKD patients [3, 111]. Since FGF23 upregulates Dkk1 and high phosphorus levels are directly correlated with FGF23, phosphorus could increase Dkk1 levels through FGF23 [2]. Phosphorus supplementation also leads to higher levels of sclerostin and Dkk1 transcripts in the tibiae of parathyroidectomized CKD rats, which suggests that the effect is likely not mediated by PTH [109, 112]. While studies have reported a link between the levels of FGF23 and sclerostin in CKD, whether one upregulates the other is currently unknown [111, 113]. As both FGF23 and sclerostin are increased very early in the disease, they could be upregulated by the same unknown factor [9, 107].

### 7.3. PTH Inhibits Sclerostin Production in CKD

Recent studies have reported a negative relationship between PTH and sclerostin. Indeed, non-CKD patients with hyperparathyroidism have consistently lower sclerostin levels than patients with normal or suppressed PTH levels [86, 114]. In CKD patients, serum sclerostin also inversely correlates with serum PTH but the sclerostin levels usually remain above those of healthy controls [115, 116]. The fact that both PTH and sclerostin are elevated in CKD may suggest that osteocytes become resistant to the suppressing actions of PTH as part of the development of skeletal PTH resistance [117] or that the unknown upregulator of sclerostin has a stronger effect in uremic condition.

### 7.4. Sclerostin, Dkk1, SFRP4, and RANKL in CKD

Apart from its recognized suppressing effect on bone formation, inhibition of the Wnt/*β*-catenin pathway has been associated with an increased RANKL/OPG ratio in non-CKD mice [28, 35, 36, 59]. Considering that Wnt inhibitors are generally upregulated in CKD, which likely contributes to suppression of the Wnt/*β*-catenin signaling in bone, they could also contribute to the increased levels of RANKL seen in hemodialysis patients [118]. Supporting this idea, in a genetic mouse model of polycystic kidney disease, Wnt/*β*-catenin pathway suppression was associated with high levels of both serum sclerostin and RANKL [107]. This is consistent with the lower RANKL/OPG ratio that was observed when *β*-catenin was constitutively activated in non-CKD mice [36] and suggests that sclerostin, by inhibiting the Wnt/*β*-catenin pathway of osteoblast-lineage cells in CKD, could also lead to increased bone resorption on top of decreased formation. Since the primary recognized role of sclerostin is to inhibit bone formation and increase bone resorption, it was suggested that high levels of sclerostin in CKD would contribute to increase bone fragility. A study by Ferreira et al. has recently shown that parathyroidectomized rats with nephrectomy-induced CKD had high levels of Dkk1 and sclerostin transcripts in their bones, which was associated with low bone formation rates and bone volume [109]. We have also observed increased Dkk1 transcription in the bones of our CKD rat model, which is associated with a decreased mineral content in the tibia (personal data). Finally, a study in a CKD mouse model reported that knocking out the SOST gene did not substantially affect bone structure, the only reported effect was being a decreased number of osteoclasts in the vertebrae, which could potentially be explained by a stimulation of RANKL by sclerostin [119]. In CKD stage 5 patients, high levels of sclerostin were associated with lower bone turnover, indicating that the Wnt inhibitors could contribute to renal osteodystrophy [120-122]. However, despite its association with a low bone turnover, sclerostin levels have also been positively associated with bone mineral density (BMD) in predialysis and dialysis patients, which may reflect the higher number of sclerostin-producing osteocytes in patients with higher BMD [4, 122-125]. Meanwhile, serum Dkk1 has been associated with a lower BMD at the femoral neck in predialysis CKD patients [4]. As of today, no studies have evaluated the role of Wnt inhibitors in predicting fracture risk in CKD patients. Serum SFRP4 has been shown to increase when serum sclerostin is progressively downregulated in a mouse model of CKD, suggesting a possible role of SFRP4 in maintaining Wnt inhibition despite suppression of sclerostin [107]. Further studies are needed to better understand the role of SFRPs in regulating bone metabolism during CKD.

### 7.5. Wnt Inhibitors and Vascular Calcification

Vascular calcification is a complex process that involves transdifferentiation of vascular smooth muscle cells into osteoblast-like cells that calcify the vessel walls due to the deposition of bone matrix and mineralization [126]. It has been hypothesized that Wnt inhibitors would have the same suppressing effect on vascular calcification as on bone formation in CKD. In fact, recent studies have reported that low serum sclerostin levels were observed in CKD patients and kidney transplant recipients suffering from arterial calcification, which could suggest a protective effect on the vessels [127, 128]. Moreover, in hemodialysis patients, high sclerostin levels have been associated with a decrease in vascular calcification [125]. In contrast, a significant number of other studies observed a positive correlation between circulating sclerostin levels and vascular and aortic valve calcification in CKD patients [129–134]. These seemingly contradictory findings could reflect a number of pathological mechanisms: (1) sclerostin inhibits osteoblastic differentiation in bone but not osteoblastic transdifferentiation in the vessels; (2) sclerostin-induced decrease in bone turnover may lead to higher circulating calcium and phosphorus levels that stimulate vascular calcification; (3) there might be a shift in the source of sclerostin production in CKD. While the circulating sclerostin produced in the bone might indeed delay the progression of vascular calcification, the calcified vasculature could itself be a major source of sclerostin alongside the bone as the disease progresses, which could explain the positive association between the two; (4) as a corollary, the role of the Wnt inhibitors during progression of CKD could be different from their role in the healthy population, which would explain the variable results in nondialyzed versus dialyzed population. Increased vascular expression of sclerostin has been reported in the calcified aortic valves of CKD patients and the aortas of mice [132, 135] and also by our own observations in thoracic aortas from our CKD rats (personal data). Expression of other Wnt inhibitors, namely, SFRP-1, SFRP-2, and SFRP-4, has also been reported in the calcified vessels of a CKD rat model [132], supporting the idea that the vasculature could be a contributor to serum Wnt inhibitors levels.

### 7.6. Sclerostin and Survival in CKD

Despite its association with bone anomalies and vascular calcification, reports on the link between serum sclerostin levels and mortality rates are conflicting. While a few studies report that dialysis patients with high serum sclerostin levels have an improved survival rate and a decreased risk of cardiovascular mortality [125, 136, 137], there are also other reports showing a positive or no association of serum sclerostin with all-cause mortality [111, 138, 139] Until now, the association between levels of Wnt inhibitors and mortality in CKD is not well defined.

## 8. Is There a Role in Targeting Wnt Pathway Inhibitors in CKD-MBD?

There is evidence that Wnt inhibitors are associated with bone turnover and mineral density anomalies in CKD and are potentially involved in the development of vascular calcification. However, their exact role still remains to be studied. Antibodies against Wnt inhibitors were proven useful to improve bone parameters in animal models of osteoporosis, fracture, and osteomalacia caused by hypophosphatemic rickets, suggesting they could also have a beneficial effect on bone in CKD [55, 56, 78, 140, 141].

### 8.1. Effects of Anti-Sclerostin and Anti-Dkk1 on Bone in CKD

A recent study by Moe et al. using anti-sclerostin antibody in a rat model of CKD induced by genetic polycystic kidney disease has yielded conflicting results. In rats with low PTH, treatment with anti-sclerostin antibodies has succeeded in increasing trabecular bone volume and mineralization, but did not significantly improve bone strength. However, these bone anabolic effects were not observed in animals with high PTH levels, indicating that these antibodies might be useful in low bone turnover state [142]. Encouraging results have also been obtained by Fang et al. with anti-Dkk1 antibodies in mild CKD diabetic mice without hyperparathyroidism. In this model, treatment with anti-Dkk1 antibodies surprisingly also decreased sclerostin levels and improved bone abnormalities [106]. The same two studies as mentioned above showed that treatments with anti-sclerostin and anti-Dkk1 antibodies were associated with a decrease in the severity of vascular calcification, reinforcing the idea that antibodies against Wnt inhibitors might be useful to prevent the onset of CKD-MBD [106, 142]. Nevertheless, treatment with these antibodies alone was not sufficient to prevent all the features of CKD-MBD, notably FGF23 elevation [106]. Simultaneous treatment with phosphate binders in order to avoid hyperphosphatemia and FGF23 elevation is therefore mandatory in order to control CKD-MBD [106]. In brief, the use of antibodies to selectively target abnormally expressed Wnt inhibitors in CKD seems therefore promising in CKD, but their efficacy and safety remain currently unknown. Furthermore, there are still concerns that inhibition of Wnt inhibitors may in fact worsen vascular calcification in human so that further studies need to be conducted before these antibodies could be used in CKD patients.  Table 1 summarizes the animal studies that have been conducted regarding anti-sclerostin and anti-Dkk1 and their effects on bone and vascular calcification.

## 9. Conclusion

Our knowledge of how the Wnt/*β*-catenin pathway is regulated and of how this regulation affects bone turnover in CKD continues to expand, allowing us to better understand the pathophysiologic mechanisms of CKD-MBD. While the handful of studies that have investigated the use of monoclonal antibodies against Wnt inhibitors in CKD yielded encouraging results, the safety of such treatment will have to be thoroughly assessed before their use can be considered in CKD patients. Mechanistic studies in animals and translational studies in humans including iliac crest biopsies will without a doubt allow us to discover new therapeutic treatments in order to improve CKD-related bone disease in the future.

## Figures and Tables

**Table 1:** Effects of targeted treatments using antibodies on bone anomalies and vascular calcification in animal models of CKD.

Treatment	Model	Effect on bone^∗^	Effect on vascular calcification^∗^	Other effects^∗^
Anti-sclerostin	Cy/+ rats with low PTH	↑ bone volume/total volume (trabecular bone) ↑ trabecular mineralization surface	↓ % of animals with signification arterial calcification	↑ bone SOST transcripts

Anti-sclerostin	Cy/+ rats with high PTH	—	—	—

Anti-Dkk1	CKD stage 2 (partial nephrectomy) diabetic mice	↑ bone formation rate ↑ bone volume ↑ trabecular number and volume ↑ osteoblast and osteoclast number	↓	↓ RunX2 in aorta ↓ circulating sclerostin ↑ sm22*α* in aorta ↑ klotho in aorta

Anti-Dkk1 + phosphate binders	CKD stage 2 (partial nephrectomy) diabetic mice	↑ bone formation rate ↑ bone volume ↑ trabecular number and volume ↑ osteoblast and osteoclast number	↓	↓ RunX2 in aorta ↓ circulating sclerostin ↑ sm22α in aorta ↑ klotho in aorta ↓ circulating FGF23

Cy/+: genetic model of polycystic kidney disease. ^∗^Effects as compared to Cy/+ with high/low PTH or CKD stage 2 diabetes without treatment.
